# Downscaling GRACE total water storage change using partial least squares regression

**DOI:** 10.1038/s41597-021-00862-6

**Published:** 2021-03-26

**Authors:** Bramha Dutt Vishwakarma, Jinwei Zhang, Nico Sneeuw

**Affiliations:** 1 School of Geographical Sciences, University of Bristol, University Road, BS8 1SS Bristol, UK; 2 Institute of Geodesy, University of Stuttgart, Geschwister-Scholl Strasse 24D, Stuttgart, Germany

**Keywords:** Hydrology, Hydrology

## Abstract

The Gravity Recovery And Climate Experiment (GRACE) satellite mission recorded temporal variations in the Earth’s gravity field, which are then converted to Total Water Storage Change (TWSC) fields representing an anomaly in the water mass stored in all three physical states, on and below the surface of the Earth. GRACE provided a first global observational record of water mass redistribution at spatial scales greater than 63000 km^2^. This limits their usability in regional hydrological applications. In this study, we implement a statistical downscaling approach that assimilates 0.5° × 0.5° water storage fields from the WaterGAP hydrology model (WGHM), precipitation fields from 3 models, evapotranspiration and runoff from 2 models, with GRACE data to obtain TWSC at a 0.5° × 0.5° grid. The downscaled product exploits dominant common statistical modes between all the hydrological datasets to improve the spatial resolution of GRACE. We also provide open access to scripts that researchers can use to produce downscaled TWSC fields with input observations and models of their own choice.

## Abstract (summary)

**Table:** 

Measurement(s)	Gravity
Technology Type(s)	gravity field theory • computational modeling technique
Factor Type(s)	geographic location • temporal interval
Sample Characteristic - Environment	water body
Sample Characteristic - Location	global

Machine-accessible metadata file describing the reported data: 10.6084/m9.figshare.13503114

## Background & Summary

GRACE based TWSC estimates have helped hydrologists and meteorologists to close the water budget, validate data products, estimate groundwater loss, study droughts and floods, monitor diminishing water bodies, and improve geophysical models^1–4^. Users can download global GRACE mass change estimates from various centres at three different levels: level 2, level 3 and time series level. Level 2 products are noisy geopotential spherical harmonic coefficients up to degree and order 96, which must be filtered and processed to obtain level 3 products usually sampled at 0.5° × 0.5° or 1° × 1° grid cell^4–6^. Computing catchment averages of level 3 monthly fields provides us with the time series of TWSC. GRACE products are an excellent dataset for studies concerning large catchments but are less effective for small scale studies^1,4,7,8^.

The ideal spatial resolution or the native resolution for the GRACE mission can be expressed as the minimum spherical distance between two resolvable Dirac pulses on the surface of the Earth, which has been shown to be ≈3° for spherical harmonic fields up to maximum degree and order of 96^8,9^. Filtering the GRACE spherical harmonic fields degrades the signal quality and the spatial resolution further^8,9^. Therefore, level 3 products must be corrected for signal damage due to filtering^5,10,11^. Mascon products are another type of level 3 GRACE products that employ constrained regularization of inter-satellite range rate to estimate localized mass change^12,13^. They are known for reducing side effects of filtering: loss in the spatial resolution and signal quality, offering better signal to noise ratio at the spatial scales of ≈90000 km^2^ ^4,13^. These high resolution mascon solutions at 0.5° or 1° grids are only interpolated samples of a coarse GRACE product, which means they are spatially correlated^14^. stated that the total energy in a mascon can be accounted for by aggregating all the mascons with a radius of 600 km and the spatial leakage errors in 1° × 1° mascons from Goddard Space Flight Center (GSFC) are equivalent to those of a gravity field filtered with a Gaussian 300 km filter. To conclude, available high resolution GRACE products do not contain physical information at a spatial scale better than the native resolution of GRACE^13^. Hence, in order to employ GRACE at finer spatial scales, we must downscale GRACE products by incorporating additional information at a higher resolution. In general, there are two broad categories of downscaling approaches: dynamical and statistical. In dynamical downscaling large-scale and lateral boundary conditions are used to realistically simulate regional features, while in statistical downscaling predictor variables ate determined that represent the statistical relationship between large-scale variable and small scale variables^15^. Both have their *pros* and *cons*, such as the former is physically based but computationally expensive and strongly dependent on boundary conditions, while the latter is computationally cheap and easy to implement but requires long and reliable observations and depends on choice of predictors and quality of input data.

It has been shown that the effective resolution can be improved by assimilating information at a better spatial resolution^16–20^. Various data assimilation techniques have been devised and successfully implemented, for example, in improving operational weather forecast, predicting ocean dynamics, and modelling soil moisture content^18,21,22^. Recently several studies have assimilated hydro-geodetic data and hydrological models to estimate, calibrate, or validate hydrological flux variables. For example: modelling river runoff with the help of hydro-geodetic approaches^23,24^, estimating catchment-scale water budget using a Kalman filter framework^25,26^, and calibration or/and validation of hydrological model outputs using GRACE^27,28^. The ensemble Kalman approach filter has been used effectively to assimilate GRACE TWSC into a Land Surface Model (LSM) to improve model performance^17,28–30^. Several non-parametric methods have also been proposed to improve spatio-temporal knowledge of hydrological variables. For example^31^, predicted ground water level changes by incorporating GRACE with hydro-meteorological variables in an Artificial Neural Network (ANN) framework.^32^ demonstrated the efficacy of ANN to predict TWSC from precipitation, soil moisture, and temperature, and^33,34^ used ANN and Machine Learning to produce high-resolution TWS estimates. Recently^35^ demonstrated that statistical downscaling can help us fill temporal gaps in GRACE data and^36^ developed a statistical downscaling approach that uses evapotranspiration data to downscale GRACE TWSC.

Inspired by recent developments in assimilating models and hydro-geodetic observations, we present a statistical downscaling approach that improves the spatial resolution of GRACE from ≈3° to 0.5° grid. The method employs a multivariate regression model that integrates multiple components of water budget (WaterGAP hydrology model (WGHM) TWSC, GRACE TWSC, several estimates of precipitation, evapotranspiration and runoff). The regression is carried out at a residual signal level obtained by removing the dominant seasonal signal and linear trend. It also accounts for time lag and lead between various water budget components. The method finds common spatiotemporal modes by employing Partial Least-squares Regression (PLR), and then uses these modes to reconstruct (redistribute) GRACE observed mass change at the spatial resolution of WGHM. We demonstrate that our method is able to learn from high resolution model TWSC and resolve spatial features such as river channels, which is not possible from conventional GRACE products. Since the information on high resolution mass change is obtained from high resolution model, the downscaled product is expected to vary with the input data. In this study our aim is to provide users with a framework that they can use to obtain better resolved TWSC estimates with datasets and models they are confident of. Hence we refrain from commenting on the best dataset on the input side because robust validation of TWSC at the grid scale is not possible. We demonstrate that the method ensures conservation of GRACE-derived mass at catchment scale for 160 catchments spread over the globe.

## Methods

Complex mechanisms drive spatiotemporal variability in hydrological flux variables, which are related to each other *via* the water budget equation. Our aim is to relate hydrological flux variables to grid scale TWSC. This can be achieved by employing a multivariate linear regression model that relates the predictand (*S*) (the signal to be predicted) to the predictor (*L*) (obtained from set of observations) as

1 $$\begin{array}{l}S=LH,\end{array}$$

where *S*(*n* × *g*) is a matrix with *n* rows, one for each epoch, and *g* columns, one for each grid cell in a river catchment. The predictor matrix *L*(*n* × *d*) has *n* rows, one for each epoch, and *d* columns containing Precipitation *P*, Evapotranspiration *ET*, Runoff *R*, and catchment average of TWSC. In other words, each column vector in *L* is a time series with *n* epochs. *H*(*d* × *g*) is the prediction matrix.

Setting up *L* efficiently is crucial for the success of (1). Since many data products are available for each water budget component and their performance varies with space and time, using multiple products for each variable provides the regression model with flexibility to rely relatively more on a dataset that offers stronger spatio-temporal common modes. Secondly, the water budget components are known to have temporal lead or lag with respect to each other^37–39^, and the dominant signal is driven by the annual water cycle. Therefore, the regression in (1) will be more efficient, if we i) expand the observation space by including *k* time shifted versions of each flux variable, and ii) operate at the residual signal level. We demonstrate this with an example: assume we have total *m* products for *P*, *ET*, *R*, and TWSC for a time period from January 2003 to December 2015. First, we expand *P*, *ET*, and *R*, to get a matrix where number of rows correspond to number of epochs and number of columns represent number of grid cells corresponding to a catchment, then we obtain *k* shifted versions of equal length time series, for example: for *k* = 12, we will obtain 12 equal length time series for each precipitation grid cell in the catchment we are interested in, where the first will start at January 2003 and end at December 2014, the second time series starting at February 2003 and ending at January 2015, and so on. Then we remove a cyclo-stationary mean from the corresponding shifted time series to obtain Δ*P*, Δ*ET*, and Δ*R*. A cyclo-stationary mean is an annual cycle that represents the mean behaviour over the observation period. ΔTWSC is the time series from GRACE with cyclo-stationary mean signal and a linear trend removed. Hence *L* becomes,

2 $$\begin{array}{cll}L & = & [\Delta P\;\Delta ET\;\Delta R\;\Delta \mathrm{TWSC}],{\rm{where}}\\ \Delta P & = & [\Delta {P}_{11}\;\Delta {P}_{12}\;\ldots \;\Delta {P}_{21}\;\Delta {P}_{22}\;\cdots \;\Delta {P}_{{m}_{p}^{g}k}],\\ \Delta ET & = & [\Delta E{T}_{11}\;\Delta E{T}_{12}\;\ldots \;\Delta E{T}_{21}\;\Delta E{T}_{22}\;\cdots \;\Delta E{T}_{{m}_{e}^{g}k}],\;{\rm{and}}\\ \Delta R & = & [\Delta {R}_{11}\;\Delta {R}_{12}\;\ldots \;\Delta {R}_{21}\;\Delta {R}_{22}\;\cdots \;\Delta {R}_{{m}_{r}^{g}k}].\end{array}$$

$\Delta {P}_{{m}_{p}^{g}k}$ is a column vector of length equal to the number of epochs *n*. ${m}_{p}^{g}$ is the product of number of precipitation products *m*_*p*_ and the number of grid cells in the catchment *g*. This means the dimension of Δ*P* will be *n* × (*m*_*p*_ × *g* × *k*). Hence the number of columns in *L* depends on the number of models for each variable $\left(m={m}_{p}+{m}_{e}+{m}_{r}+1\right)$, the number of grid cells in the region of interest, and the number of time-shifts *k*.

Equation (1) represents the ideal case, but in reality measurements suffer from noise. Therefore, the multivariate regression model in (1) becomes

3 $$\begin{array}{l}S=LH+E.\end{array}$$

Dimension reduction is crucial for multivariate regression analysis, which we achieve by using Partial Least Squares Regression (PLR), a non-parametric filtering technique developed by^40^. It decomposes the signal while minimizing the noise and preserving the mutual linear variability of measurements and unknown signals^40,41^. In other words, PLR aims to regress on those Principal Components (PCs) of measurements that highly correlate with the target signal^42,43^. Similar to Canonical Correlation Analysis (CCA), PLR obtains the PCs *via* Singular Value Decomposition (SVD) of the covariance matrix *C*_*LS*_ between predictors and predictands^41,44,45^.

In the context of this study, *S* is obtained from a hydrology model that simulates TWSC at a higher spatial resolution compared to GRACE. For a given *L*, we can obtain the covariance matrix *C*_*LS*_(*d* × *g*), which can be decomposed using SVD:

4 $$\begin{array}{l}{C}_{LS}={L}^{T}S={U}_{C}\;{\Sigma }_{C}\;{V}_{C}^{T},\end{array}$$

where *U*_*C*_(*d* × *r*) and *V*_*C*_(*r* × *g*) are joint normalized eigenvectors for *L* and *S*, which are also called the canonical modes, and Σ_*C*_(*r* × *r*) is a diagonal matrix containing covariance between *L* and *S*. *r* is the number of canonical modes from SVD, obtained as the rank of covariance matrix *C*_*LS*_^43^. The PCs of *L*, which are significantly correlated with *S*, can be obtained by projecting *L* on *U*_*C*_ to get *U*_*L*_(*n* × *r*):

5 $${U}_{L}=L\,{U}_{C}.$$

Hence, we can write $L={U}_{L}{U}_{C}^{T}$, which can be substituted in (3):

6 $$S={U}_{L}\;{U}_{C}^{T}\;H\;+E,$$

which can also be written as

7 $$S={U}_{L}\,K+E,$$

where *K*(*r* × *g*) is the transformed regression matrix obtained by projecting *H* on *U*_*c*_. Since we do not expect the total mass change in a catchment to change after downscaling, we can use the mass conservation as a constraint:

8 $$S={U}_{L}\,K+E,\;{\rm{subject}}\,{\rm{to}}\;S{A}_{w}=\Delta {M}_{{\rm{GRACE}}}.$$

where *A*_*w*_ is the area vector for grid cells belonging to the catchment and Δ*M*_GRACE_ is the catchment average of TWSC from GRACE. Using (7) and (5) in the constraint, we get

9 $$\begin{array}{ccc}S & = & {U}_{L}\,K+E,\;{\rm{subject}}\,{\rm{to}}\;L\,{U}_{C}\,K\,{A}_{w}=\Delta {M}_{{\rm{GRACE}}},\\ S & = & {U}_{L}\,K+e,\;{\rm{subject}}\,{\rm{to}}\;L\,{U}_{C}\,K=\frac{\Delta {M}_{{\rm{GRACE}}}\,{A}_{w}^{T}}{| {A}_{w}^{2}| }.\end{array}$$

Bringing the constraint in the observation space and solving for *K* using the least squares method,

10 $$\begin{array}{lcl}\widehat{K} & = & {({X}^{T}X)}^{-1}{X}^{T}Y,{\rm{where}}\\ X & = & \left[\begin{array}{c}{U}_{L}\\ {U}_{L}\end{array}\right],{\rm{and}}\\ Y & = & \left[\begin{array}{c}S\\ \frac{\Delta {M}_{{\rm{GRACE}}}{A}_{w}^{T}}{| {A}_{w}^{2}| }\end{array}\right]\end{array}$$

*U*_*L*_(*n* × *r*) constitutes the PCs. $\widehat{K}(r\times p)$ is the reformed regression matrix that can be combined with *U*_*C*_ to obtain the optimal prediction matrix $\widehat{H}$,

11 $$\widehat{H}={U}_{C}\,\widehat{K}.$$

This concludes the training part, where we obtained a prediction matrix from known *S* and *L*. The prediction matrix can now be used to estimate the predictand *S*, that is, estimates of TWSC at a higher spatial resolution. Since we have regressed at the residual level, we can obtain the full downscaled product by restoring the part of TWSC signal that was removed earlier. To summarize, the statistical downscaling using PLR has five major steps:

1. arrange global data products to obtain time series vectors representing hydrological flux variables,
2. obtain dominant modes of variability between observations and a high resolution hydrology model,
3. estimating the prediction matrix from selected canonical modes,
4. transforming the prediction matrix from mode space to signal space, and
5. obtaining the downscaled GRACE product.

### Step by step implementation

**Fig. 1:**
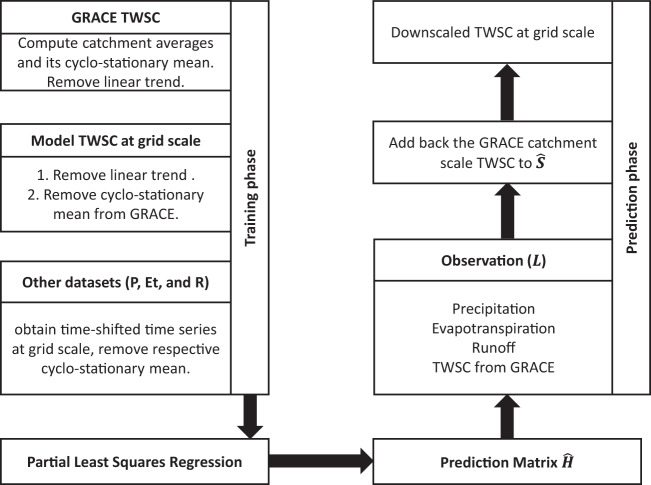
Flowchart of the downscaling approach.

A conceptual flow chart of the process is provided in Fig. 1. The first step is to obtain datasets of *P*, *ET*, *R*, and TWSC. Usually the global products are available in grid cell format at different spatial resolutions. We choose *P*, *ET*, and *R*, at the same spatial resolution as WGHM. GRACE TWSC catchment averages are computed using the following relation,

12 $${f}_{{\rm{c}}}=\frac{1}{{A}_{{\rm{c}}}}\mathop{\int }\limits_{\Omega }\,f(\theta ,\lambda )R(\theta ,\lambda ){\rm{d}}\Omega ,$$

wherein *f*_c_ is the catchment average of a global gridded field *f*(*θ*, *λ*). *R*(*θ*, *λ*) is the catchment mask with value 1 inside the catchment and 0 outside. Ω represents the domain of the Earth’s surface, *θ* and *λ* are co-latitude and longitude, and dΩ is the infinitesimal surface element sin *θ*d*θ*dλ.

A pre-processing step is performed to subtract the dominant signals and retain the residuals from the data. First we shift grid scale time series for *P*, *ET*, *R* by *k* = 6. We choose *k* = 6 because we are confident of capturing seasonal lead and lag with this value. If readers are confident of capturing time lead or lag between budget components with a different *k*, then they can use a different value. After obtaining time-shifted vectors, we remove a cyclo-stationary mean from the corresponding vector to obtain Δ*P*, Δ*ET*, and Δ*R*. Hydrology model based estimates of TWSC have been shown to underestimate linear trends compared to GRACE observations^46^, thus, we remove the linear trend from TWSC. Here we have two TWSC estimates, one from the model at the grid scale and another from GRACE at the catchment scale. Let us represent the detrended TWSC time series from GRACE as *M*_GRACE_ and its cyclo-stationary mean by ${\widetilde{M}}_{{\rm{GRACE}}}$. The residual $\Delta {M}_{{\rm{GRACE}}}$ is obtained by removing the cyclo-stationary mean from detrended GRACE TWSC $\Delta {M}_{{\rm{GRACE}}}={M}_{{\rm{GRACE}}}-{\widetilde{M}}_{{\rm{GRACE}}}$. The predictand matrix *S* consists of TWSC grid cells values from WGHM. The time series for each grid cell is first detrended and then the cyclo-stationary GRACE TWSC signal is removed, $S={M}_{{\rm{WGHM}}}-{\widetilde{M}}_{{\rm{GRACE}}}$. Please note that the GRACE TWSC estimates are obtained at catchment scale, and therefore, the same value is subtracted from every grid cell. Hence, the observations from GRACE, *P*, *ET*, and *R* are regressed on the difference between WGHM and GRACE. Therefore, the dominant part of TWSC from GRACE is maintained.

13 $$\begin{array}{lcl}S & = & LH,\\  & = & [\Delta P\,\Delta ET\,\Delta R\,\Delta {M}_{{\rm{GRACE}}}]{[{H}_{P}{H}_{ET}{H}_{R}{H}_{M}]}^{T}.\end{array}$$

*H* is unknown while *L* and *S* are known.

In the next step, we compute *C*_*LS*_ and decompose it by SVD to get *U*_*C*_(*d* × *r*) and *V*_*C*_(*r* × *g*). *r* is the number of canonical modes from SVD and it can attain a maximum value that is the rank of covariance matrix *C*_*LS*_. In this study we choose *r* = 10, because including more modes does not affect the efficacy of PLR method^20^. We can obtain *U*_*L*_ as

14 $${U}_{L}=L\,{U}_{C}.$$

Now we have all the components to implement equations in (10) and obtain $\widehat{K}$. This leads to determination of the prediction matrix $\widehat{H}$,

15 $$\widehat{H}={U}_{C}\,\widehat{K}.$$

$\widehat{H}$ can then be used to predict $\widehat{S}=L\;\widehat{H}$. The full downscaled product is obtained by adding back the linear trend and the cyclo-stationary mean signal to $\widehat{S}$.

## Data Records

**Fig. 2:**
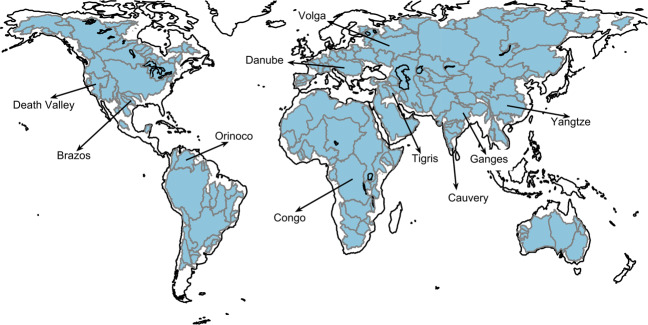
160 catchments under investigation. We have labelled names of randomly selected 10 catchments just for illustration.

We use GRACE Level 3 mascon products from Jet Propulsion Laboratory (JPL) and GRACE level 2 spherical harmonic coefficients provided by the Institute of Geodesy, Graz University of Technology (IFG)^13,47,48^. We use precipitation (*P*) datasets from three centres (CPC, DELAWARE, and GLDAS NOAH025 M 2.1)^49–53^, two model based estimates of evapo-transpiration (*ET*) products (GLDAS, SeB)^53–55^, and two model based runoff (*R*) estimates (GLDAS and MERRA)^53,56,57^. We implement the method for 160 river catchments, where the smallest catchment is the Negro river basin in Uruguay with an area of 62518 km^2^ and the largest catchment is the Amazon river basin with an area of 4672876 km^2^. The catchment boundaries have been downloaded from GRDC^58^ (*cf* Fig. 2). The prior model information is obtained from WGHM, which is a global water resource and use model that simulates water flows among all relevant continental water storage compartments, including canopy, snow, soil, groundwater, lakes, reservoirs, rivers and wetlands. Despite the complex yet realistic model setup, the uncertainties in climate forcing limit the accuracy of WGHM in monitoring large-scale water storage variation^27,46,59,60^. We obtained model TWSC at a spatial resolution of 0.5° × 0.5° for a period from January 2003 to December 2016^59–61^. Each dataset spans at least from January 2003 to December 2015. The data used in this study have been summarized in the Table 1.

**Table 1:** Datasets used.

Dataset	centre	spatial resolution
*Precipitation	CPC	0.5° × 0.5°
	DELAWARE	0.5° × 0.5°
	GLDAS NOAH025 M 2.1	0.5° × 0.5°
*Evapotranspiration	GLDAS NOAH025 M 2.1	0.5° × 0.5°
	SeB MOD16	averaged to 0.5° × 0.5°
*Runoff	GLDAS NOAH025 M 2.1	0.5° × 0.5°
	MERRA	0.5° × 0.5°
model TWSC	WGHM	0.5° × 0.5°
*GRACE TWSC	JPL mascon, ITSG	3° × 3°
	ITSG	≈65000 km^2^

Using these dataset we obtain downscaled TWS fields, which are available to users on figshare as netcdf files with four variables: Lat, Long, time and EWH_mm^62^. Lat and Long are latitude and longitude vectors of dimension 259200 × 1, representing the centre of a 0.5° × 0.5° grid cell on the surface of the Earth. EWH_mm is the TWS change in terms of mm Equivalent Water Height (EWH) for that grid cell with dimensions 259200 × 144, and time is a column vector of dimension 144 × 2 with year and month.

## Technical Validation

### Results

We demonstrate the method for two GRACE products: level 2 spherical harmonic coefficients from ITSG and the JPL mascon products. We do not endorse these products over other, we have just chosen one mascon solution and one spherical solution for demonstrative purposes. Furthermore, the difference between various GRACE product is not huge at catchment scale, therefore, choice of GRACE data is not critical. For the spherical harmonic product, coefficient *C*_2.0_ is replaced by more accurate estimates from satellite laser ranging and the missing degree 1 terms have been replaced by degree 1 coefficients estimated by^63^. The Glacial Isostatic Adjustment (GIA) signal is removed using the ICE-6G forward GIA model^64^. Since the spherical harmonic coefficients are noisy, we filter them with a Gaussian filter of 400 km radius. Filtering affects the signal quality *via* attenuation and leakage^11^. Therefore, we use the data-driven method of deviation to repair the signal damage due to filtering^65^. The data-driven method of deviation has been shown to provide accurate mass change estimates for catchments larger than ≈65000 km^2 ^^8^. The JPL GRACE mascon solutions do not need additional corrections and are available at sampling of 0.5° × 0.5° while their effective resolution is 3° × 3°^13^. We use 10 PCs to reconstruct the signal for 160 catchments from January 2004 to December 2015.

**Fig. 3:**
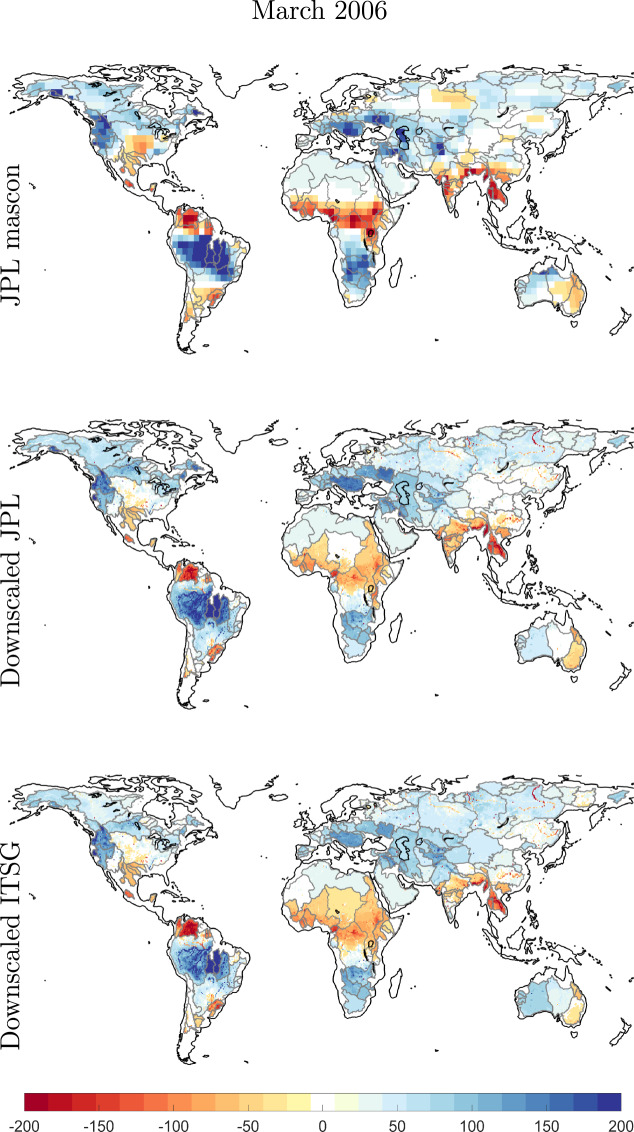
Maps of TWSC in terms of Equivalent Water Height (EWH) in units of mm for the month of March 2006. The top row contains the JPL 3° mascon product, the second row contains the downscaled product derived from these mascons, and the last row contains the downscaled product derived from data-driven leakage corrected ITSG spherical harmonics.

**Fig. 4:**
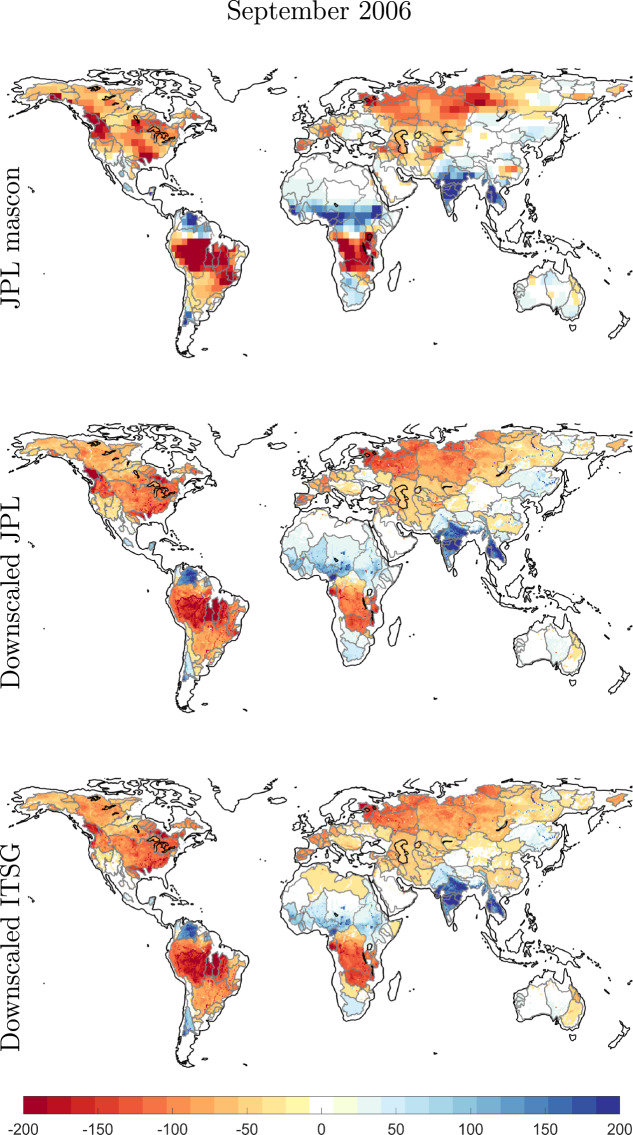
Same as for Fig. 3, but for the month of September in the year 2006.

**Fig. 5:**
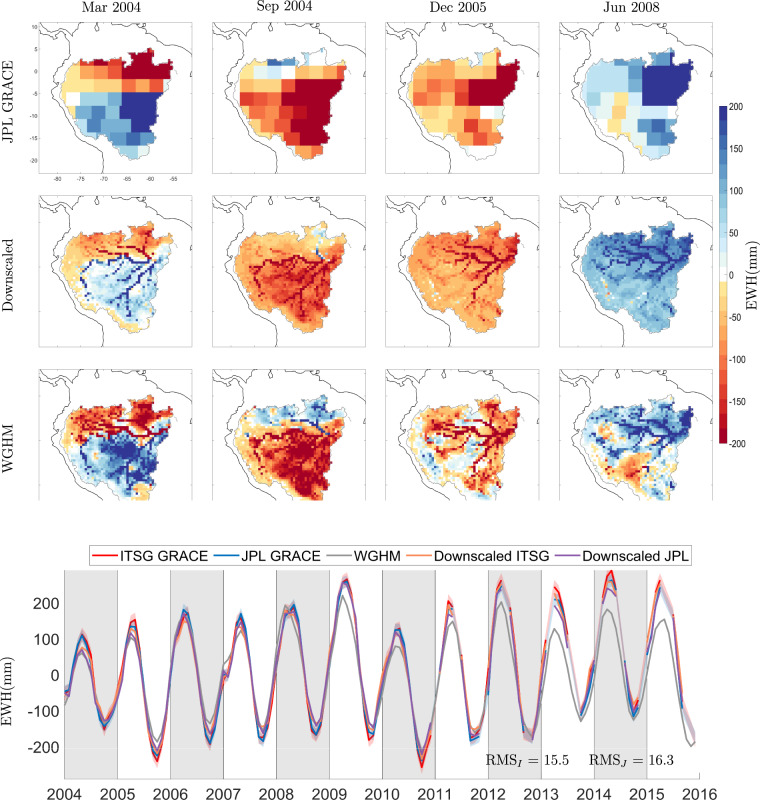
Maps of TWSC in terms of Equivalent Water Height (EWH) in units of []mm over the Amazon catchment at different points in time. The first row contains JPL 3° mascon products, the second row contains the downscaled product derived from JPL mascons, and the last row contains the WGHM model. The last figure shows catchment averages of Equivalent Water Height (EWH) from different GRACE products and the WGHM model. The RMS of difference between time series from GRACE and downscaled product is written in the time series plot. RMS_I_ corresponds to ITSG GRACE product while RMS_J_ represents JPL mascon product.

We show the coarse JPL mascon EWH maps and the downscaled maps for the month of March 2006 in Fig. 3 and for September 2006 in Fig. 4. The Year 2006 was chosen arbitrarily, months March and September are six months apart and thus would show us out of phase TWSC maps. We did not use any interpolation scheme. We can see mass change following physical water bodies^60,61^, hence, the downscaled product is able to capture spatial features better than original GRACE product. This is further demonstrated for the Amazon catchment in Fig. 5, where we plot the TWSC maps from JPL GRACE mascon, downscaled products, and the WGHM model, for four selected months. We also plot the time series of catchment average for Amazon. It is clear that the downscaled product is able to deliver mass change estimates at a higher spatial resolution.

### Validation

**Fig. 6:**
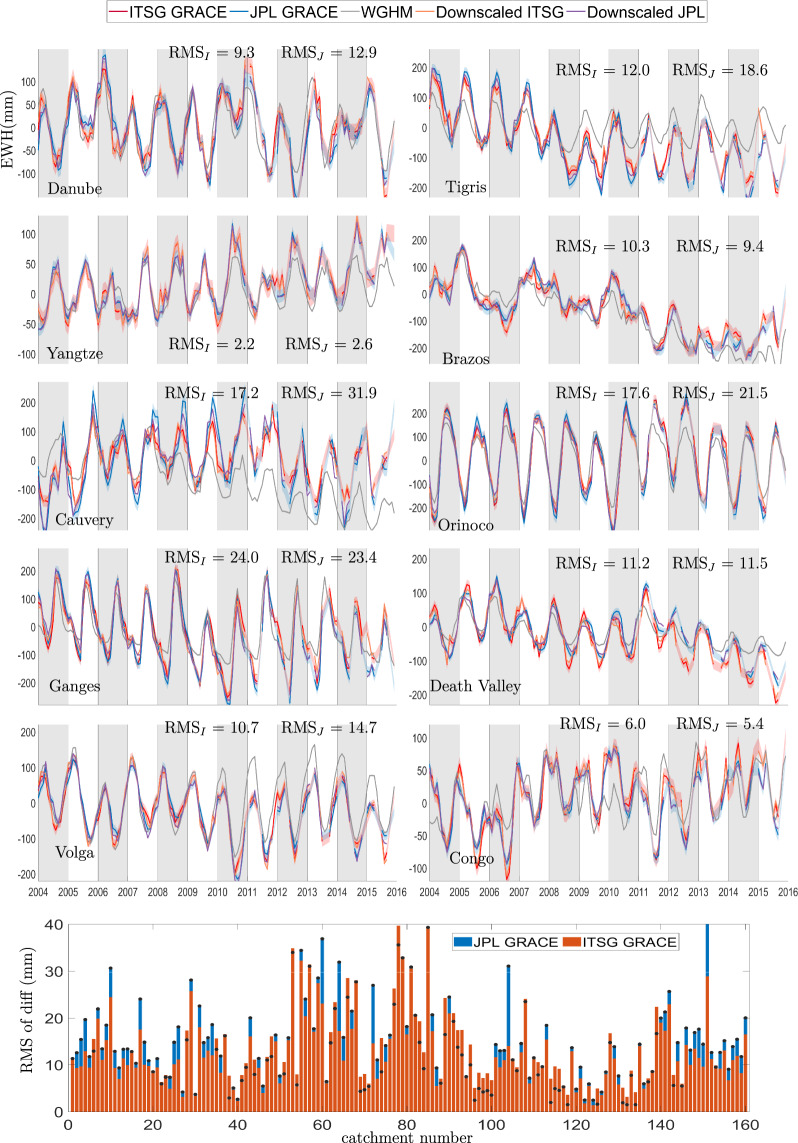
Catchment averages of Equivalent Water Height (EWH) in units of []mm from different GRACE products and the WGHM model for 10 catchments labelled in Fig. 2. The plot at the bottom of the figure shows the RMS error of difference between time series from GRACE and downscaled product over 160 catchments. The RMS of difference between catchment averages of JPL GRACE product and the corresponding downscaled product is denoted by RMS_J_, while for ITSG GRACE product is RMS_I_.

Validating gridded downscaled TWSC for the 160 catchments is not possible as no other observational dataset is available for direct comparison. Therefore, we validate the efficacy of downscaled product by checking the conservation of mass at catchment scale. In Fig. 6, we show time series for 10 catchments of various shape, size, and climatic characteristics. We plot the Root Mean Square (RMS) of difference between the TWSC time series from GRACE and downscaled products for all the 160 catchments, which represents the error introduced by the downscaling process. The RMS of the processing error is almost always smaller than the GRACE error that is typically around 20 to 30 mm^4^.

**Fig. 7:**
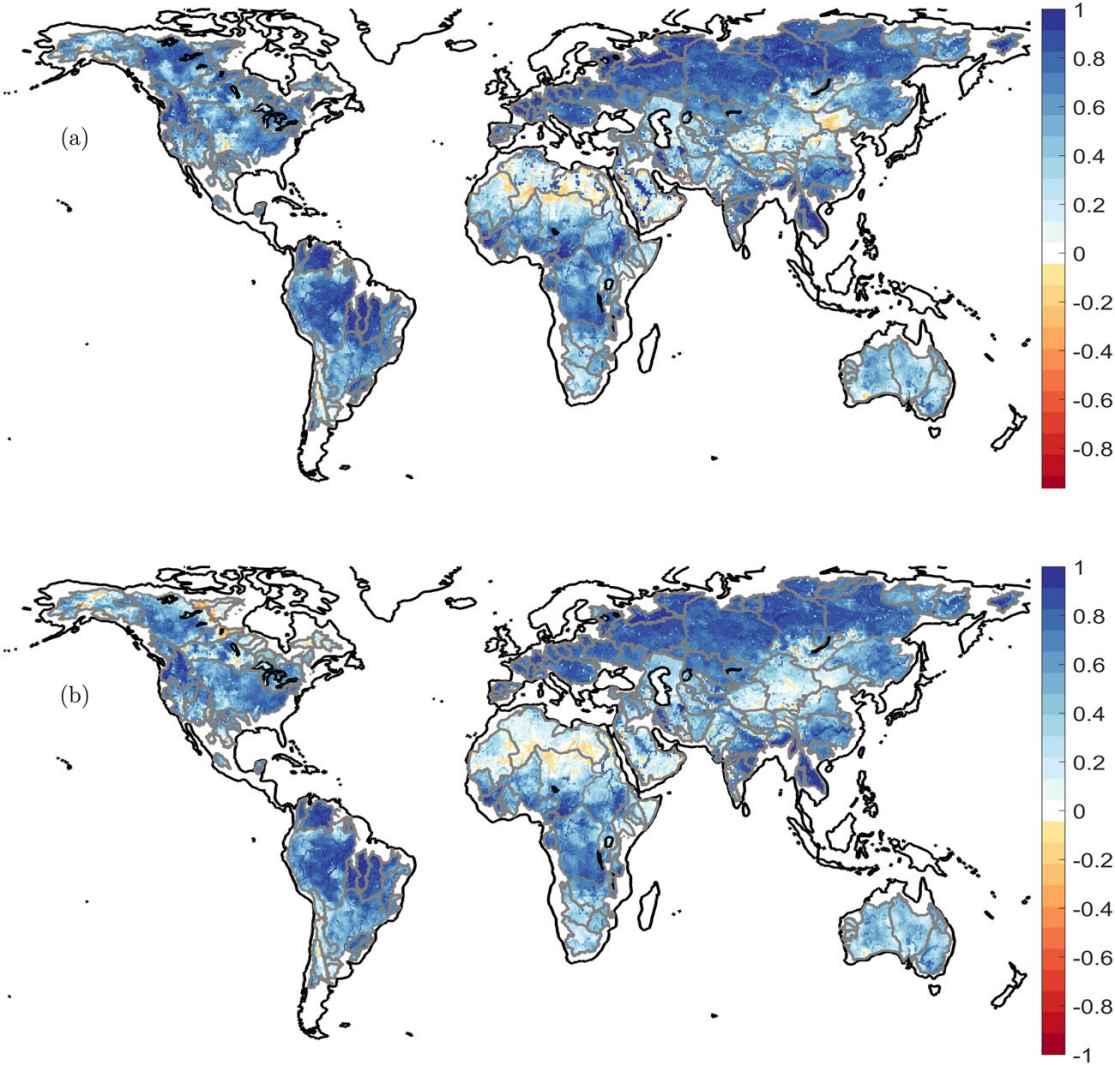
Maps of correlation between WGHM time series and downscaled TWSC time series from JPL GRACE product (**a**) and from ITSG GRACE product (**b**). There is a high correlation between high resolution WGHM product and the downscaled product, which demonstrates that the small scale features in downscaled product are driven by the WGHM model.

In Figs. 5 and 6, we compare catchment averages of GRACE products and their downscaled versions, along with the TWSC from WGHM model. A first observation is that the model simulations are not able to match GRACE observations, the difference is more prominent for catchments with poor data quality or availability, for example the Cauvery, the Tigris, or the Volga. Secondly, the downscaled product does not pick signal amplitude information from WGHM model. The catchment averages of downscaled TWSC match with the corresponding GRACE product. The water mass is redistributed to reflect additional information on river channels and landscape properties (*cf*. Figure 5). The spatial correlation between high resolution WGHM time series and the downscaled products is shown in Fig. 7, which shows that WGHM plays a significant role in redistributing the water mass change from GRACE. Together from Figs. 5–7, we can safely conclude that WGHM informed the spatial redistribution and not the signal amplitude while the principle of conservation of mass is not violated. We have provided the RMS of the process error for downscaled GRACE from JPL solution (RMS_j_) and ITSG (RMS_I_) solution on the time series plot for 160 river catchments. These catchments are distributed all over the globe (*cf* Fig. 2) and the RMS of error is small for all of them. Therefore, we can safely conclude that the efficacy of the downscaling approach is not region-dependent.

Please note that we do not claim that the downscaled product corresponds to the ground truth, as the output is only as accurate as the information from the input datasets. If users find other models or datasets more plausible, we recommend them to use the Octave/MATLAB script for generating a downscaled product on their own. The methodology uses dominant modes of variability between observations and a high resolution hydrology model to obtain downscaled product. Analysis of the prediction matrix could help us understand the relative contribution of individual input data. In Supplementary Fig. 1 we show the relative percentage contribution from dominant input datasets for one grid cell (0.5° × 0.5°) in the Amazon catchment. We do not go into detailed analysis of the prediction matrix in this study as it is an enormous task as there are 1530 grid cells in the Amazon catchment alone (the prediction matrix for the Amazon river basin alone is 64261 by 1530). Analyzing prediction matrix and understanding influence of input dataset will be a future project.

## Usage Notes

The scripts and the output data are available for download. The datsets for *P*, *ET*, *R*, and TWSC should be prepared by the user following the instructions in the ReadMe file provided along with scripts and dataset. After you have Octave/MATLAB Data files for each variable, run the script *statistical_downscaling_grids_TWS.m* in command line following the instruction in ReadMe file. The command window will ask the user to select relevant files. These will then be used to obtain a downscaled product. A downscaled dataset from January 2004 to December 2015, and an example dataset to guide users is also available.

## Supplementary information

Supplementary figure 1

## Data Availability

We have used Octave/MATLAB for processing the data. Along with the downscaled GRACE data, we also provide the script freely available for download from figshare 10.6084/m9.figshare.c.5054564^62^.
