# Supplementary figures and images for: Downscaling GRACE total water storage change using partial least squares regression
Source: Sci Data. 2021 Mar 26;8:95. doi: 10.1038/s41597-021-00862-6 (PMC7998002; doi:10.1038/s41597-021-00862-6)

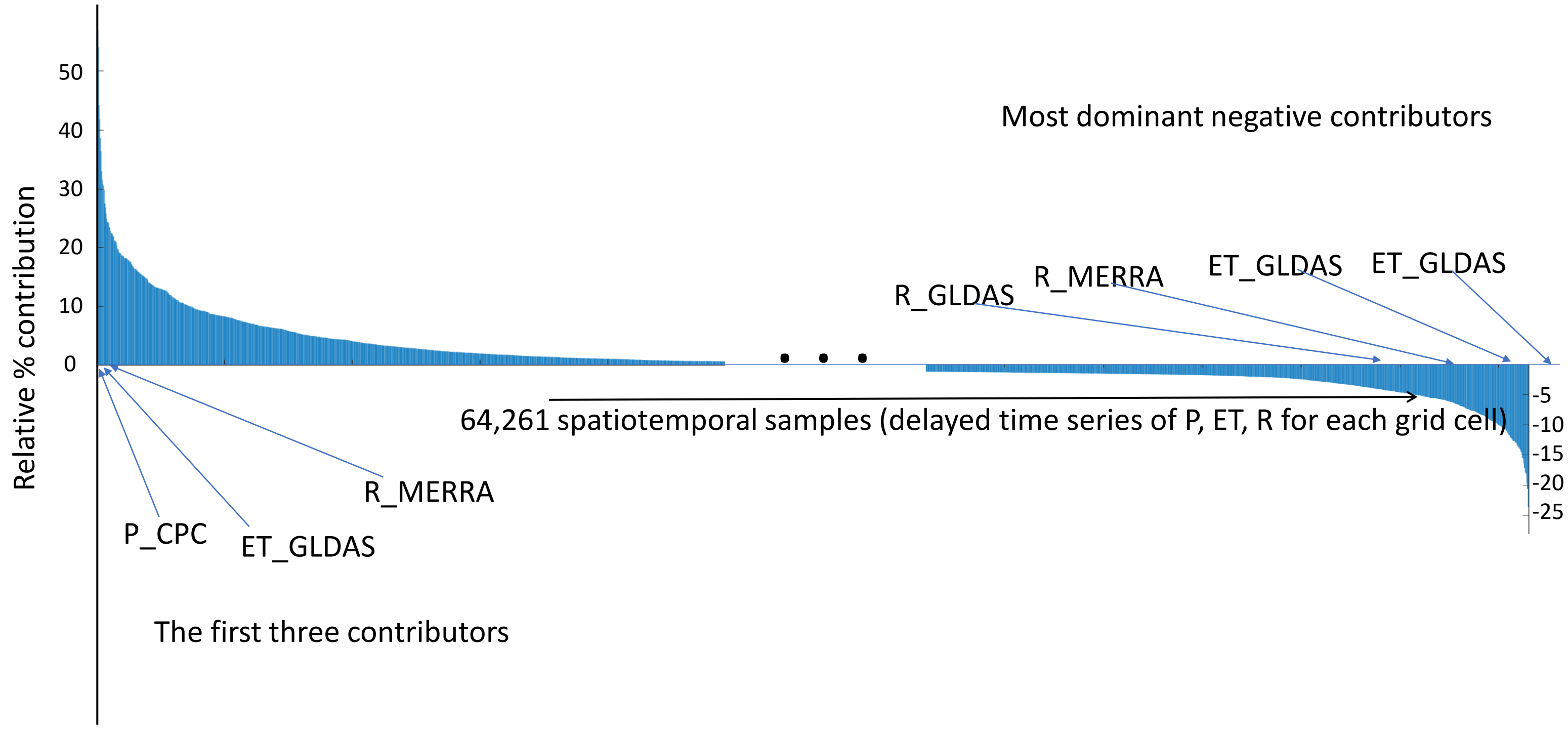

Supplement: Supplementary file 1 — Supplementary figure 1 [file 41597_2021_862_MOESM1_ESM.pdf]
